# Evaluation of Mud Worm (*Polydora* spp.) Infestation in Cupped (*Crassostrea gigas*) and Flat Oyster (*Ostrea edulis*) Broodstocks: Comparison between Magnetic Resonance Imaging and Computed Tomography

**DOI:** 10.3390/ani14020242

**Published:** 2024-01-12

**Authors:** Livio Galosi, Fabrizio Dini, Marina C. T. Meligrana, Lorenzo Gennari, Elena Tamburini, Alessandra Roncarati

**Affiliations:** 1 School of Biosciences and Veterinary Medicine, University of Camerino, Via Circonvallazione 93/95, 62024 Matelica, MC, Italy; fabrizio.dini@unicam.it (F.D.); marina.meligrana@unicam.it (M.C.T.M.); alessandra.roncarati@unicam.it (A.R.); 2 Bi.vi. S.r.l., 62012 Civitanova Marche, MC, Italy; gennari.bivi@gmail.com; 3 Department of Environmental and Prevention Sciences, University of Ferrara, Via L. Borsari 46, 44121 Ferrara, FE, Italy; tme@unife.it

**Keywords:** advanced diagnostic techniques, marine bivalves, parasitic infestation, shell damages

## Abstract

**Simple Summary:**

The parasites of the genus *Polydora* spp. cause great damage to oyster broodstocks, both Cupped (*Crassostrea gigas*) and Flat oysters (*Ostrea edulis*), as they excavate channels in the shell of the mollusk, forming mud blisters. The infestation negatively affects the oysters’ welfare, as it influences respiratory activity, induces oxidative stress in the animal, and impacts the productivity of the farm, as heavily parasitized mollusks cannot be commercialized. With the aim of obtaining a non-invasive assessment of the presence of this parasite, the use of two diagnostic methods, magnetic resonance imaging and X-ray computed tomography, was investigated. These methods have been shown to be effective in diagnosing the infestation, and although the current costs of their application are prohibitive for production reality, the possibility of analyzing or evaluating remediation techniques on large stocks of animals offers interesting perspectives.

**Abstract:**

The Polichete worms of the genus *Polydora* are considered very destructive for oysters, excavating channels in their shell and inducing oysters to create mud blisters in response to the irritation, interfering with their physiology and ethology. The parasite also causes important economic damage for oyster farmers, as products with a high degree of infestation cannot be commercialized. The present study aims to evaluate whether two non-invasive advanced diagnostic techniques, computed tomography scans (CT) and magnetic resonance imaging (MRI), are suitable to show the alterations induced by this parasite on live *Crassostrea gigas* and *Ostrea edulis* oyster broodstocks. A CT scan is also able to identify small lesions in the shell during the first stage of infection. MRI allows for the visualization of the advanced status of the lesions when blisters occupy the inner surface of the shell and can impact the health status and the economic value of the mollusk. Both techniques resulted in satisfactory spatial resolution, and no motion artifacts were reported, thus enabling the authors to faithfully visualize in vivo the damage caused by the parasite.

## 1. Introduction

In the middle Adriatic Sea, along the Italian coast, both Cupped (*Crassostrea gigas*) and Flat oyster (*Ostrea edulis*) can be successfully raised in offshore long-line farms [1]. As grow-out practices are well-assessed, more attention has been paid to the end-product quality, with particular regard to processing and packaging after harvesting. Most of the shellfish farmers sell their products directly to wholesalers in 20 kg bags because of limited equipment and space on board and a lack of land-based facilities. The challenge is to assess farming practices aimed at obtaining the best end-product quality in a cost-effective way in the shortest possible time according to a sustainable approach [2]. The breeding of oysters in eutrophic waters with a high level of organic enrichment can be hindered and/or compromised by parasitic infections. In fact, the shells of bivalve mollusks, particularly those of oysters, serve as an underlayer for innumerable species of benthic organisms, which live either on the surface of the shells or perforate them and colonize the mollusks from the interior [3].

Polychaetes, and in particular those belonging to the *Polydora* genus, are worms commonly associated with mud blisters in bivalve shellfish, mainly oysters [3,4,5,6,7,8]. The infestation was reported by the beginning of the nineteenth century, causing mud blisters in the shell and sometimes being associated with mortality [9]. The parasites use the shells as substrates to construct burrows, causing modifications to the internal and external surfaces of the shells [10]. Tunnels can cause weak points where shells may crack during packaging or transportation [11]. Worm infestation affects the inner shell presentation and reduces the commercial value of the oysters, creating unsightly mud blisters that are unappealing to consumers [12]. Furthermore, *Polydora* spp. interferes with *Crassostrea gigas* physiology and ethology, modifying the behaviour and respiratory physiology of the mollusks and inducing oxidative stress. This leads to a decrease in oyster growth, depending on the intensity of the infestation [13,14]. Therefore, apart from the damage caused to the shell, infestation by *Polydora* spp. gives the oyster a watery appearance and impoverishes the organoleptic characteristics, leading to a diminished value of the product [15,16]. Indexes to evaluate the damage induced by *Polydora* spp. were proposed, taking into consideration a visual examination of the inner surface of the valves (presence of tunnels and/or chambers) [17].

In any case, the investigation and analysis of the inner surface of the valves and soft tissues in marine bivalves classically relied on destructive methods since a shell completely encloses the animal. In fact, anatomical structures are generally studied by means of histological sections after the opening and dissection of the flesh from the shell [18,19]. Despite providing much valuable information, these standard methods have the disadvantage of being time-consuming and necessarily damaging, preventing subsequent samplings of the same individuals. Moreover, the examination of biochemical and physiological changes requires the sampling of a large amount of tissue and inevitably involves the euthanasia of numerous specimens.

In the veterinary sciences, diagnostic techniques are available to obtain detailed images of different body areas, like magnetic resonance imaging (MRI) and computed tomography (CT). The MRI is a diagnostic technique that uses magnetic fields to obtain detailed images of all body areas. This test does not use ionizing radiation, and for this reason, it is possible to undergo the procedure several times, even at close intervals. CT is a diagnostic technique used in radiology that uses ionizing radiation to obtain three-dimensional images. During the examination, the areas of the body to be studied are crossed by a beam of X-rays, which, thanks to a sophisticated processing system, will be transformed into three-dimensional images that allow the radiologist to formulate the diagnosis.

Previous studies on MRI reported that this technique was able to quantify the volume and mass of the whole flesh and of some organs in the cupped oyster [20,21,22] and in the flat oyster [23]. However, there are too few studies comparing different non-destructive techniques to determine the damage caused by the *Polydora* worm.

The present study aims to test and evaluate the suitability of two non-invasive advanced diagnostic techniques, CT and MRI, to show the alterations induced by *Polydora* spp. infection in the shell of live *Crassostrea gigas* and *Ostrea edulis* oyster broodstocks.

## 2. Materials and Methods

### 2.1. Animals

The experiments were carried out on 20 Cupped oyster (*Crassostrea gigas*) broodstocks, weighing 89.5 ± 14 g, and on 20 Flat oyster (*Ostrea edulis*) broodstocks, weighing 68.8 ± 7 g. All the specimens came from a 2-month finishing period at a long-line farm located in the middle Adriatic Sea. They were randomly collected and harvested from natural beds situated in front of Porto Recanati (Marche Region, Italy; 3–5 nautical miles offshore). The broodstocks had not been anesthetized and were analyzed individually. 

### 2.2. Advanced Diagnostic Techniques

A computed tomography (CT) scan was performed on the animals using a GE CT-E device (GE HealthCare Device, Milan, Italy). 1 × 1 mm^2^ transverse sections of the broodstocks were carried out. Tomographic images were processed in order to obtain a multiplanar reconstruction using Horos^TM^ medical software (https://horosproject.org/, accessed on 9 January 2024, Horos Project, Annapolis, MD, USA). 

Magnetic resonance imaging (MRI) was subsequently carried out on the same animals using a Vet-MR 0.2 Tesla (Esaote, Genoa, Italy). Turbo 3D-T1, T2-weighted, and STIR scans were performed, allowing for the three-dimensional reconstruction of the mollusk and the differentiation of its organs and structures. 

### 2.3. Macroscopic Findings

To verify the correspondence of channels and blisters excavated by *Polydora* spp. with the images acquired via the diagnostic instruments, the oysters were euthanized, and the shell was cut with an electric saw.

## 3. Results

### 3.1. Advanced Diagnostic Techniques

**Figure 1:**
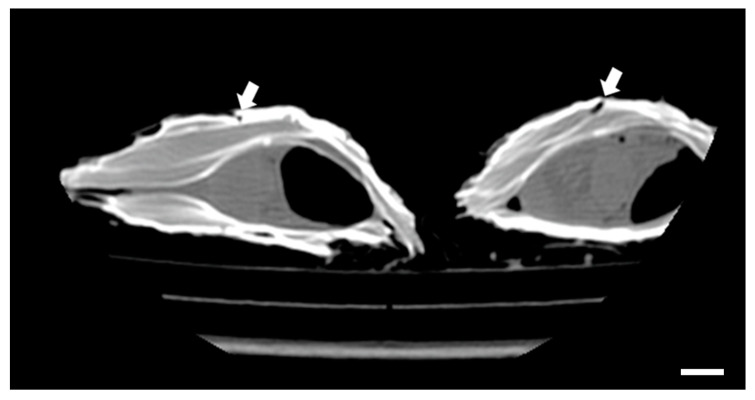
Computed tomography scan of Pacific oysters (*C. gigas*), sagittal reconstruction. Note the presence of channels excavated by the parasites *Polydora* spp. (arrows). Scale bar = 2.5 cm.

Both techniques resulted in satisfactory spatial resolution, and no motion artifacts were reported, thus enabling the authors to faithfully visualize the damage caused by the parasite. On CT scans, small channels excavated by the parasite in the shell were seen in all the specimens (*n* = 40). These images showed the structure of the shell in minute detail and, in particular, the presence of channels caused by the polychaetae worm *Polydora* spp. (Figure 1).

**Figure 2:**
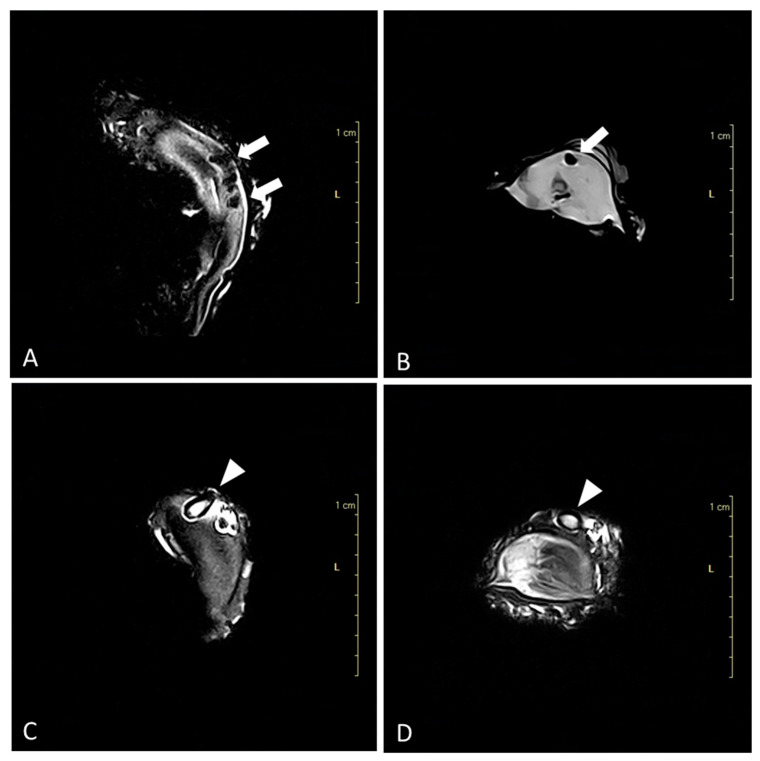
Magnetic resonance imaging. (**A**) Flat oyster (*O. edulis*), dorsal Turbo 3D-T1 weighted scan; blisters containing necrotic material are identified on the inner surface of the shell (arrows). (**B**) Cupped oyster (*C. gigas*), sagittal Turbo 3D-T1 weighted scan; blister is identified on the inner surface of the shell (arrows). (**C**) Flat oyster (*O. edulis*), dorsal Turbo 3D-T1 weighted scan; the parasite of the genus *Polydora* spp. is identified in the blister (arrowhead). (**D**) Cupped oyster (*C. gigas*), sagittal Turbo 3D-T1 weighted scan; the parasite of the genus *Polydora* spp. is identified in the blister (arrowhead).

On MRI, blisters with necrotic material were seen in Cupped and Flat oysters (Figure 2A,B). The high resolution of the images enabled the authors to clearly visualize the mollusks and differentiate between their main components: the mantle in the posterior section, the gills in the ventral section, the visceral mass in the anterior section, and the adductor muscle in the central part. The best sequence for the evaluation of the whole organism was the Turbo 3D-T1 weighted scans studied in the three spatial planes because it provided a better resolution of the hard and soft components of the shellfish. In some cases (*n* = 7 in Cupped oysters and *n* = 12 in Flat oysters), the parasite was identified inside the blisters as it showed a hyperintense structure (Figure 2C,D). 

The CT scan was able to identify channels in the shell, while MRI was more indicated for identifying blisters and burrows containing necrotic material on the inner surface of the shell. 

### 3.2. Macroscopic Findings

**Figure 3:**
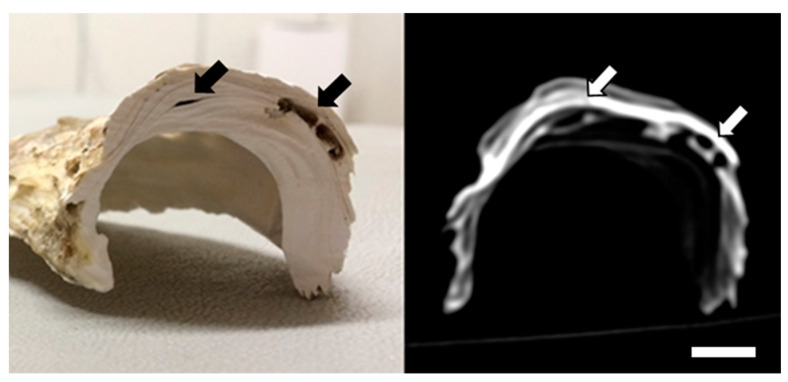
Comparison between macroscopic section and computed tomography imaging (bone window) of Cupped oyster (*C. gigas*) shell. Note the presence of channels excavated by the parasites *Polydora* spp. (arrows). Scale bar = 1 cm.

**Figure 4:**
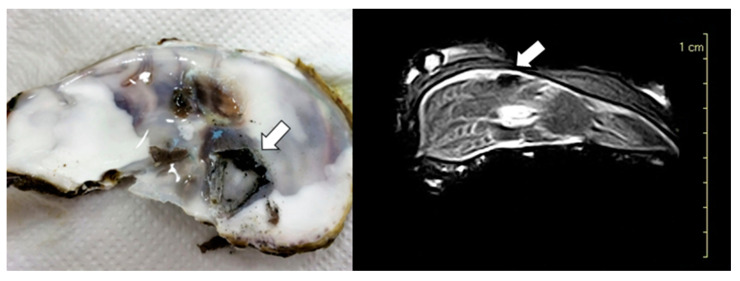
Comparison between macroscopic section and MRI of Flat oyster (*O. edulis*) shell. The mud blister excavated by the parasites *Polydora* spp. was identified (arrows).

In all cases (*n* = 40), the correspondence between macroscopic lesions and CT (Figure 3) and MRI imaging (Figure 4) was confirmed.

## 4. Discussion

In the present study, the results demonstrated that the two tested advanced diagnostic techniques, MRI and CT scanning, are able to evaluate alterations induced by *Polydora* spp. without damaging the broodstocks. Neither method was invasive or destructive, therefore making it possible to monitor and individually measure certain characteristics of the broodstocks over time without having to sacrifice the oyster. For this reason, during the summer, oyster farmers along the coast of the northern Gulf of Mexico use desiccation to treat oysters for mud blister worms. Nevertheless, stocking density or ploidy was considered unlikely to be effective in preventing mud blister worm infestation [24].

As reported in the literature [14], worms of the genus *Polydora* spp. induce oysters to create mud blisters in response to irritation within their shells, interfering with oyster physiology and ethology. Ascertaining the presence of this worm is important, particularly if the broodstocks come from the wild. It is essential for shellfish farmers to be aware of the presence of the parasite because, in this case, they can perform different treatments against *Polydora* infestation and, subsequently, obtain a reduction in blister formation. These treatments are particularly successful when a recirculating aquaculture system for land-based plants is available [25]. Cold storage, together with land-based wet storage facilities, represent the key factors in making the product available when harvesting is impossible due to bad weather or when toxic algae are in bloom. These invertebrates, being filter feeders, could potentially compete with cultivated oysters for phytoplanktonic resources. They can also reduce the water flow through the culture and restrict food availability. Blisters can heavily impact the internal organs of the mollusks, causing atrophy of the adductor muscle. The latter is also highlighted in a study in which *Polydora* affected the production of gametes due to the lesions that were formed adjacent to the gonads [26]. The lack of identification of the species of the parasite represents a limiting factor in this study. Therefore, further studies are needed in order to verify whether different species of the genus *Polydora* exert a diverse pathogenic action in oysters.

From this study, it emerged that CT and MRI were suitable to be employed in different ways. A CT scan proved more appropriate for assessing the damage to the shell, especially when compared with the macroscopic assessment. Indeed, a CT scan was also able to identify small lesions in the shell during the first stage of infection. The presence of blisters increases the fragility of the shell, which becomes more prone to breaking during the processing of the product. This parasite causes an important economic loss to oyster farmers since the final product becomes unsuitable for the market [27,28]. MRI allowed for the visualization of the advanced status of the lesions when blisters occupy the inner side of the shell and can impact the health status and the economic value of the mollusk. According to a previous study [29], there is a highly significant linear correlation between the results obtained from MRI and those from histology. The use of MR imaging and CT scanning also proved to be highly valuable tools in determining the condition index of live oysters [30]. 

## 5. Conclusions

By means of the MRI in the current study, it was possible to assess the alterations caused by the *Polydora* spp. parasites on the shellfish, such as the atrophy and/or the detachment of the adductor muscle. These two advanced diagnostic techniques represent an innovative step forward in oyster farming, providing a harmless means of selecting oyster broodstocks, offering substantial benefits in the field of veterinary research, and opening up many perspectives for further studies. Routine application of these instruments will depend on their cost; although still expensive, the price of MRI and CT scans is beginning to go down due to the increase in their use for small animals and to the development of devices dedicated to them. The evaluation of a small number of subjects can, however, give a very useful indication of the degree of parasite infestation of the stock in its entirety. In future studies, the application of advanced diagnostic techniques will make it possible to quantify the lesions and verify the possibility of repairing the shell tissue and the feasibility of the healing methods.

## Data Availability

All data are included in the manuscript.
